# Maternal vitamin D status, gestational diabetes and infant birth size

**DOI:** 10.1186/s12884-017-1600-5

**Published:** 2017-12-15

**Authors:** Helena H. Hauta-alus, Heli T. Viljakainen, Elisa M. Holmlund-Suila, Maria Enlund-Cerullo, Jenni Rosendahl, Saara M. Valkama, Otto M. Helve, Timo K. Hytinantti, Outi M. Mäkitie, Sture Andersson

**Affiliations:** 1 Children’s Hospital, University of Helsinki and Helsinki University Hospital, Biomedicum 2 C, P.O. Box 705, 00020 HUS Helsinki, Finland; 2 Folkhälsan Research Center, Haartmaninkatu 8, P.O.Box 63, 00014 Helsinki, Finland; 3 Center for Molecular Medicine, Karolinska Institute and Clinical Genetics, Karolinska University Hospital, 171 76 Solna, Stockholm Sweden

**Keywords:** Maternal vitamin D status, Newborn vitamin D status, 25-hydroxy vitamin D concentration, Gestational diabetes mellitus, Birth size, Birth weight, Birth length, Head circumference, Ponderal index

## Abstract

**Background:**

Maternal vitamin D status has been associated with both gestational diabetes mellitus (GDM) and fetal growth restriction, however, the evidence is inconsistent. In Finland, maternal vitamin D status has improved considerably due to national health policies. Our objective was to compare maternal 25-hydroxy vitamin D concentrations [25(OH)D] between mothers with and without GDM, and to investigate if an association existed between maternal vitamin D concentration and infant birth size.

**Methods:**

This cross-sectional study included 723 mother-child pairs. Mothers were of Caucasian origin, and infants were born at term with normal birth weight. GDM diagnosis and birth size were obtained from medical records. Maternal 25(OH)D was determined on average at 11 weeks of gestation in pregnancy and in umbilical cord blood (UCB) at birth.

**Results:**

GDM was observed in 81 of the 723 women (11%). Of the study population, 97% were vitamin D sufficient [25(OH)D ≥ 50 nmol/L]. There was no difference in pregnancy 25(OH)D concentration between GDM and non-GDM mothers (82 vs 82 nmol/L, *P* = 0.99). Regression analysis confirmed no association between oral glucose tolerance test results and maternal 25(OH)D (*P* > 0.53). Regarding the birth size, mothers with optimal pregnancy 25(OH)D (≥ 80 nmol/L) had heavier newborns than those with suboptimal pregnancy 25(OH)D (*P* = 0.010). However, mothers with optimal UCB 25(OH)D had newborns with smaller head circumference than those with suboptimal 25(OH)D (*P* = 0.003), which was further confirmed as a linear association (*P* = 0.024).

**Conclusions:**

Maternal vitamin D concentration was similar in mothers with and without GDM in a mostly vitamin D sufficient population. Associations between maternal vitamin D status and birth size were inconsistent. A sufficient maternal vitamin D status, specified as 25(OH)D above 50 nmol/L, may be a threshold above which the physiological requirements of pregnancy are achieved.

**Trial registration:**

The project protocol is registered in ClinicalTrials.gov in November 8, 2012 (NCT01723852).

**Electronic supplementary material:**

The online version of this article (10.1186/s12884-017-1600-5) contains supplementary material, which is available to authorized users.

## Background

Vitamin D deficiency, defined as a circulating 25-hydroxy vitamin D (25(OH)D) concentration below 50 nmol/L, has been common among Finnish pregnant women [1]. However, due to recent changes in national health policies, intake of vitamin D has increased resulting in decreasing rates of vitamin D deficiency [2–5]. Vitamin D deficiency has been associated with gestational diabetes mellitus (GDM) [6], but the evidence is inconsistent [7, 8]. Of all pregnancies, 1–14% are affected by GDM [9], and globally GDM prevalence has been increasing in line with increasing obesity [10]. GDM is the most common pregnancy complication in Europe [11]. In Finland, the prevalence of GDM has increased from 6% in 2008 to 11% in 2014 [12]. GDM increases the risk of adverse pregnancy and neonatal outcomes, and the risk of obesity, metabolic syndrome, diabetes, and cardiovascular disease in later life both of the mother and the child [13].

Fetal growth may have later health implications also within the normal-birth-weight range [14]. Poor maternal vitamin D status has been related to fetal growth restriction [15] but it is unknown whether maternal vitamin D status associates with birth size in infants with normal birth weight.

Many of the findings regarding the relationship between vitamin D deficiency and GDM are based on case-control studies, which may include a potential selection bias. Case-control studies often focus on high-risk groups, for example women who are overweight and sedentary, which are independent risk factors for vitamin D deficiency as well (for example [16]).

The objectives of the present study were to compare 25(OH)D concentration at two consecutive time points between mothers with and without GDM, and to investigate associations between maternal factors and infant’s birth size, and the potential role of 25(OH)D concentration therein.

The study is part of the longitudinal Vitamin D Intervention in Infants (VIDI) study.

## Methods

### Recruitment and study participants

At Kätilöopisto Maternity Hospital, Helsinki, Finland, 987 families were recruited into the VIDI study between January 2013 and June 2014, after delivery during the mother’s hospital stay. According to the inclusion criteria, the mothers were of Caucasian origin without regular medication and with singleton pregnancy. Exclusion criteria for the newborns were: nasal continuous positive airway pressure treatment >one day, intravenous glucose infusion, seizures, duration of phototherapy >three days and need for nasogastric tube >one day. The infants were born between 37 + 0 and 42 + 0 weeks of gestation, and newborn’s birth weight was appropriate for gestational age (SD-score [SDS] between −2.0 and +2.0). Of the recruited eligible families, 29% (987/3408) agreed to participate in the VIDI study. For the present cross-sectional study, we included mothers who had a record from a community prenatal clinic or baseline questionnaire and both two maternal 25(OH)D measurements. Two infants with a congenital disease (Down syndrome and Rieger syndrome) were excluded. Thus, the total number of subjects was 723. Of the infants, 367 were girls and 356 boys. Number of subjects in each analysis is reported in Tables.

### 25(OH)D analyses

Maternal pregnancy serum samples for 25(OH)D measurements were collected at community prenatal clinics at gestational weeks 7 to 25 between June 2012 and November 2013 as part of the mothers’ normal follow-up [hereafter referred to as pregnancy 25(OH)D]. At birth, umbilical cord blood (UCB) for 25(OH)D measurement was obtained at gestational weeks 37 to 42 between January 2013 and May 2014 [hereafter referred to as UCB 25(OH)D]. Both pregnancy serum and UCB plasma 25(OH)D were analysed simultaneously using the IDS-iSYS fully automated immunoassay system with chemiluminescence detection (Immunodiagnostic Systems Ltd., Bolton, UK). Detailed information on 25(OH)D analysis has been previously reported [5]. The quality and accuracy of the serum 25(OH)D analysis is validated on an ongoing basis by participation in the vitamin D External Quality Assessment Scheme (DEQAS, Charing Cross Hospital, London, UK).

Both 25(OH)D concentrations were corrected by applying a linear regression equation (Oct 2014 value (nmol/L) = [(early 2014 value) – 8.2] / 0.99) provided by the manufacturer because of methodological changes in the IDS-iSYS system between 2014 and 2016 (see Additional file 1). We re-analysed a subsample of 77 samples and verified the correction (adjusted R^2^ = 0.922, SEE = 9.2 nmol/l).

We employed UCB 25(OH)D to reflect both the maternal vitamin D status at the end of pregnancy and the newborn’s vitamin D status at birth [1]. We defined vitamin D deficiency as 25(OH)D < 50 nmol/L, and vitamin D sufficiency as 25(OH)D ≥ 50 nmol/L, since a concentration of ≥50 nmol/L is considered sufficient for bone health [17]. Suboptimal vitamin D status was defined as 25(OH)D < 80 nmol/L, and optimal vitamin D status as 25(OH)D ≥ 80 nmol/L, as has been suggested based on calcium absorption studies [18].

### Maternal and newborn data

Maternal data were obtained from a self-administered baseline questionnaire, filled in after delivery, and from medical records. Maternal height (cm) and weight (kg) before pregnancy and parity were collected primarily from the prenatal maternity card or, if missing, from our baseline questionnaire. Gestation was determined by first trimester ultrasound examination. Maternal age was determined at delivery. Parity was categorised into nullipara, secundipara and multipara (>two deliveries). Prepregnancy body mass index (BMI) (kg/m^2^) was categorised into underweight (<18.5), normal weight (18.5–24.9), overweight (25.0–29.9) and obese (>30.0).

Prepregnancy weight and weight recorded in prenatal clinics were utilised to calculate gestational weight gain (GWG) (kg). We recorded GWG at first measurement, at approximately the 12th, 20th and 30th gestational weeks, and at last measurement. GWG was adjusted for consecutive gestational week. Concerning the analysis of total GWG, absolute values were used, and women who had their final weight recorded more than three weeks before the delivery were omitted (*n* = 7). Total GWG was categorised into inadequate, adequate and excessive based on national recommendations by prepregnancy BMI: recommended GWG for underweight mothers was 12.5–18.0 kg, for normal weight 11.5–16.0 kg, for overweight 7.0–11.5 kg, and for obese 5.0–9.0 kg [19].

Education level was graded from one (=comprehensive school/lower secondary education) to six (university degree/first or second stage of tertiary education). Education was re-categorised into ‘lower’ and ‘higher’ education (lower = lower or upper secondary or post-secondary non-tertiary education, higher = first or second stage of tertiary education), due to a low number of subjects in other education categories. Prepregnancy smoking status was assessed as number of cigarettes per day. Maternal use of supplements, specific brand names, dosing, and date of commencement were recorded. We calculated the average daily intake of vitamin D from supplementation during the last two months of pregnancy.

Birth size, including birth weight (kg), length (cm), and head circumference (cm), was measured by midwives according to standard procedure. These data and the duration of pregnancy were retrospectively collected from birth records. Birth size measures were transformed into SDS by using Finnish sex-specific normative data for fetal growth [20]. Ponderal index was calculated (birth weight (kg) / birth length (m)^3^) and standardised into sex-specific z-score.

### Assessment of GDM

The diagnosis of GDM was based on a two-hour 75 g oral glucose tolerance test (OGTT). According to the national guidelines, GDM was diagnosed if the OGTT results exceeded cut-offs for one or more values: fasting plasma glucose ≥5.3 mmol/l, 1-h ≥ 10.0 mmol/l and 2-h ≥ 8.6 mmol/l [19]. An OGTT was performed at gestational weeks 10 to 40 between October 2012 and March 2014, and the results were collected from prenatal maternity cards or the hospital laboratory database. In general, screening for GDM depends on a presence of risk factors according to national recommendations [19] and based on these, OGTT was performed on 490 (54.5%) of the participating mothers. None of the pregnant women in our study received insulin therapy nor other regular medication, but mothers with GDM obtained dietary counselling at community prenatal clinics [19].

### Statistical analysis

The normality of the variables was visually inspected. Outliers (*n* = 18) of 25(OH)D concentrations were identified with Normal probability plot of residuals, Leverage and Cook’s Distance diagnostic tests, and omitted from the analyses. Season with four categories affected maternal 25(OH)D concentrations. Thus, season at pregnancy blood sampling and at birth was coded using dummy variables (with autumn as a reference) in ANCOVA and used as a covariate.

The data included partially missing information. Imputation of missing values for education (*n* = 12) and parity (*n* = 2) were conducted using the median value in subgroups by GDM status. Missing data on prepregnancy smoking as number of cigarettes daily were imputed as a median value (= zero) by GDM status according to smoking status (*n* = 22). Imputation of missing values for GWG at 12 gestational weeks was conducted using a mean value of two consecutive measurements (*n* = 12). Missing values of other variables were not imputed. Maternal characteristics in Table 1 are described only as un-imputed values.

**Table 1:** Maternal characteristics in GDM and non-GDM mothers. *P*-values refer to differences between the groups

Maternal characteristics	*n*	Non-GDM	*n*	GDM	*p* value
Age at delivery (y)	642	31.4 ± 4.3	81	32.7 ± 4.5	0.018
Level of education^a^	632	5.0 ± 1.3	79	4.7 ± 1.4	0.051
Parity	640	1.5 ± 0.7	81	1.5 ± 0.7	0.275
Prepregnancy smoking, number of cigarettes daily	625	1.2 ± 3.7	76	2.0 ± 4.6	0.064
Alcohol consumption before pregnancy, portion/wk.	625	1.9 ± 2.0	78	2.3 ± 3.5	0.755
Prepregnancy height (cm)	642	166.3 ± 6.0	81	165.9 ± 5.4	0.398
Prepregnancy weight (kg)	631	63.5 ± 10.2	81	72.1 ± 13.5	<0.001
Prepregnancy BMI (kg/m^2^)	639	23.0 ± 3.5	81	26.2 ± 4.8	<0.001
Duration of gestation at OGTT (wk)	323	26.3 ± 4.3	80	25.9 ± 5.9	0.774
Duration of gestation at pregnancy blood sampling (wk)	642	11.3 ± 1.9	81	11.2 ± 2.2	0.089
Duration of gestation at delivery (wk)	642	40.2 ± 1.1	81	40.1 ± 1.1	0.410
Pregnancy 25(OH)D (nmol/L)	642	81.9 ± 19.5	81	80.0 ± 21.2	0.417
UCB 25(OH)D (nmol/L)	642	80.1 ± 20.0	81	78.4 ± 18.8	0.448
Supplemental vitamin D intake during pregnancy (*μ*g/d)	621	15.5 ± 16.6	76	13.5 ± 10.8	0.162
Cumulative gestational weight gain at^b^ (kg)					0.093
first measurement	580	1.7 ± 0.5	74	1.6 ± 0.5	
12th gestational week	580	3.8 ± 1.0	74	3.6 ± 1.1	
20th gestational week	580	6.3 ± 0.8	74	6.2 ± 0.8	
30th gestational week	580	9.7 ± 0.5	74	9.7 ± 0.4	
last measurement	580	13.8 ± 0.4	74	13.7 ± 0.3	

*GDM* gestational diabetes mellitus, *25(OH)D* 25-hydroxy vitamin D, *UCB* umbilical cord blood

Values are means ± SD

^a^Scale from 1 = lower secondary education to 6 = first or second stage of tertiary education

^b^Values are adjusted for duration of gestation

Independent sample t-tests, Mann-Whitney U-tests, repeated measures ANOVA or the Pearson Chi-Square test, when appropriate, were applied to compare maternal characteristics between GDM and non-GDM mothers. The difference in 25(OH)D between GDM and non-GDM mothers was investigated with ANCOVA adjusted for season, maternal age, education and prepregnancy BMI. Association between pregnancy 25(OH)D and OGTT results/birth weight, and between UCB 25(OH)D and head circumference at birth were tested with univariate linear regression. Prevalence of vitamin D deficiency in non-GDM and GDM mothers were tested with Fisher’s Exact test.

Newborn birth size was investigated in categories of maternal prepregnancy BMI (underweight, normal weight, overweight, obese), GWG (inadequate, adequate, excessive), prepregnancy smoking status (yes, no), maternal education (higher, lower), parity (nullipara, secundipara, multipara), GDM status (yes, no), vitamin D status (suboptimal, optimal) in pregnancy and in UCB, with ANCOVA with Bonferroni correction when applicable, and adjusted for maternal height. Changing covariates appearing in the ANCOVA models were: prepregnancy BMI, GWG at last measurement, smoking, education, parity, GDM and 25(OH)D concentrations. Using both 25(OH)D concentrations as covariates in the GWG analysis induced a multicollinearity problem based on Cook’s Distance and Levene’s test, but excluding these covariates from the model did not change the results.

Results are shown as means or adjusted means with SD or SEM. The means and medians were similar in both vitamin D concentrations. Associations were considered significant at *P* < 0.05. All statistical analyses were conducted using the IBM SPSS program for Windows, version 22 (IBM, Chicago, IL, USA).

## Results

In the study population 97% were vitamin D sufficient [25(OH)D > 50 nmol/L] in both mothers and newborns, while optimal vitamin D status [25(OH)D above 80 nmol/L] was seen in 52% of the mothers and 45% of the newborns. Majority (95%) of the mothers took vitamin D supplements during pregnancy, most of them were highly educated (75% had at least a bachelor level education), and 73% had normal weight before pregnancy.

### Vitamin D status and GDM

In our cohort GDM was present in 81 of the 723 women (11%). The comparison of maternal characteristics of GDM and non-GDM mothers is summarised in Table 1. Pregnancy 25(OH)D concentration was measured on average at 11 weeks of gestation, and GDM was diagnosed on average at 26 weeks of gestation in both GDM and non-GDM mothers. Mothers with GDM were older (*P* = 0.018), heavier and had higher prepregnancy BMI (*P* for both <0.001), and their educational level was at borderline of significance (*P* = 0.051) lower compared with mothers without GDM. No differences between the groups were observed in several other characteristics, including GWG, duration of gestation, and supplemental vitamin D intake.

**Fig. 1:**
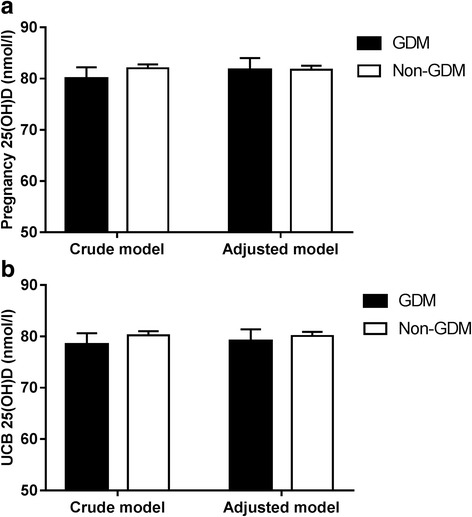
Maternal vitamin D status and gestational diabetes mellitus. Adjusted mean ± SEM values of pregnancy 25(OH)D (**a**) and UCB 25(OH)D concentration (nmol/L) (**b**) in GDM and non-GDM mothers. Adjustments are for season, maternal age, education and prepregnancy BMI. Maternal 25(OH)D concentrations were similar between non-GDM and GDM mothers. Abbreviations: 25(OH)D, 25-hydroxy vitamin D; UCB, umbilical cord blood; GDM, gestational diabetes mellitus

We investigated whether pregnancy 25(OH)D concentration differed between mothers with (*n* = 81) and without GDM (*n* = 639) (Fig. 1) in a crude model and in a model adjusted for season, maternal age, education and prepregnancy BMI. Adjusted analysis confirmed no difference in mean ± SEM pregnancy 25(OH)D concentration between GDM and non-GDM mothers (81.7 ± 2.3 vs 81.7 ± 0.8 nmol/L, *P* = 0.99). Similarly in UCB, the adjusted analysis showed no difference in 25(OH)D concentrations in infants born to women with and without GDM (79.1 ± 2.3 vs 80.1 ± 0.8 nmol/L, *P* = 0.69) (Fig. 1). These results remained when only those who had undergone OGTT were included: pregnancy and UCB 25(OH)D concentrations were similar in GDM (*n* = 80) and non-GDM mothers (*n* = 323) (*P* = 0.94 and *P* = 0.43, respectively).

We further tested for possible associations between pregnancy 25(OH)D concentration and OGTT results with a univariate regression model. We observed no association between pregnancy 25(OH)D and fasting plasma glucose (B -0.00; 95% CI -0.00, 0.00; *P =* 0.54), 1-h glucose (B 0.00; 95% CI -0.01, 0.01; *P =* 0.53) or 2-h glucose (B -0.00; 95% CI -0.01, 0.01; *P* = 0.54).

The prevalence of vitamin D deficiency [25(OH)D < 50 nmol/L] during pregnancy was similar between mothers with or without GDM [4.9% (4/81) vs 3.3% (21/642), *P* = 0.51]. However, more GDM mothers were vitamin D deficient at the delivery compared with non-GDM mothers [7.4% (6/81) vs 2.8% (18/642) (*P* = 0.042)]. However, of these six deficient GDM-mothers five (83%) were smokers, and correspondingly in non-GDM mothers four out of eighteen were smokers (24%) (*P* = 0.018).

### Maternal 25(OH)D and newborn birth size

**Fig. 2:**
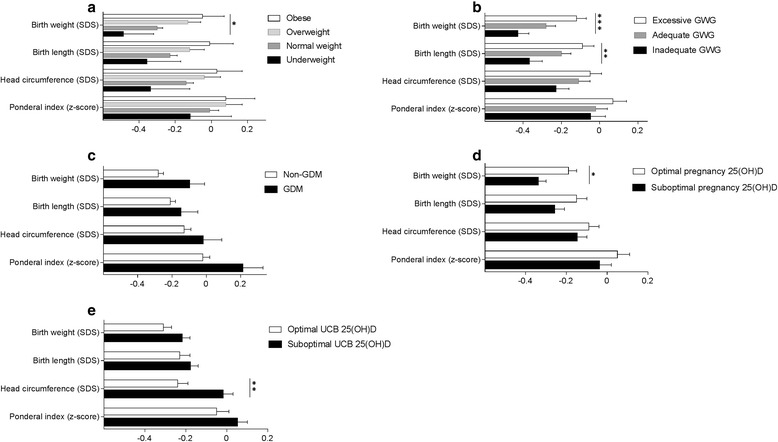
Maternal factors and birth size. Adjusted mean ± SEM values of birth weight, length, head circumference SD-scores and ponderal index z-score between (**a**) maternal prepregnancy BMI groups of underweight, normal weight, overweight and obese; (**b**) mothers’ inadequate GWG, adequate GWG and excessive GWG; (**c**) GDM and non-GDM mothers; (**d**) suboptimal and optimal pregnancy 25(OH)D; and (**e**) suboptimal and optimal UCB 25(OH)D. Statistical significance is denoted by *P < 0.05, **P < 0.01 and ***P < 0.001. Abbreviations: GWG, gestational weight gain; GDM, gestational diabetes mellitus; 25(OH)D, 25-hydroxy vitamin D; suboptimal, UCB, umbilical cord blood; 25(OH)D < 80 nmol/L; optimal 25(OH)D ≥ 80 nmol/L

The effect of maternal factors on birth size in fully adjusted models is presented in Table 2 and Fig. 2. Neither pregnancy 25(OH)D nor UCB 25(OH)D concentrations were related to birth length or ponderal index. As compared to mothers with suboptimal pregnancy 25(OH)D, mothers with optimal pregnancy 25(OH)D had heavier newborns (*P* = 0.010), but this positive association was not verified in linear regression (B 0.00; 95% CI -0.00, 0.01; *P =* 0.16). Newborn head circumference was larger in those with suboptimal UBC 25(OH)D compared with mothers with optimal UCB 25(OH)D (*P* = 0.003). Further, linear regression confirmed the inverse association between UCB 25(OH)D and head circumference (B -1.74; 95% CI -3.25, −0.23; *P =* 0.024). These results did not change after adjusting for mode of delivery (vaginal, vacuum assisted or caesarean section). Prepregnancy BMI, GWG, and parity had independent effects on birth size (Table 2 and Fig. 2).

**Table 2:** Independent effect of maternal factors on birth size^a^

	Birth weight (SDS)		Birth length (SDS)	Head circumference (SDS)^b^	Ponderal index (z-score)^c^
Maternal factors	*n*	*p* value	*p* value	*p* value	*p* value
Prepregnancy BMI		**0.020**	0.201	0.364	0.719
Underweight	19	−0.49 ± 0.17	−0.36 ± 0.19	−0.34 ± 0.22	−0.12 ± 0.23
Normal weight	494	−0.30 ± 0.03	−0.23 ± 0.04	−0.14 ± 0.04	−0.01 ± 0.05
Overweight	117	−0.13 ± 0.07	−0.12 ± 0.08	−0.04 ± 0.09	0.08 ± 0.09
Obese	44	−0.05 ± 0.12	−0.01 ± 0.13	0.03 ± 0.14	0.08 ± 0.16
Gestational weight gain		**<0.001**	**0.006**	0.186	0.505
Inadequate	164	−0.43 ± 0.06	−0.37 ± 0.07	−0.23 ± 0.07	−0.05 ± 0.08
Adequate	261	−0.28 ± 0.05	−0.20 ± 0.05	−0.11 ± 0.06	−0.02 ± 0.06
Excessive	242	−0.12 ± 0.05	−0.09 ± 0.06	−0.05 ± 0.06	0.07 ± 0.07
Prepregnancy smoking		**0.021**	0.227	0.093	0.173
Yes	101	−0.43 ± 0.08	−0.30 ± 0.09	−0.27 ± 0.10	−0.13 ± 0.11
No	573	−0.23 ± 0.03	−0.18 ± 0.04	−0.09 ± 0.04	0.03 ± 0.04
Maternal education		0.972	0.670	0.721	0.634
Higher	513	−0.26 ± 0.03	−0.19 ± 0.04	−0.11 ± 0.04	−0.00 ± 0.05
Lower	161	−0.26 ± 0.06	−0.23 ± 0.07	−0.14 ± 0.08	0.04 ± 0.08
Parity		**<0.001**	**<0.001**	**0.007**	**0.027**
Nullipara	431	−0.40 ± 0.04	−0.32 ± 0.04	−0.20 ± 0.05	−0.05 ± 0.05
Secundipara	185	−0.03 ± 0.05	−0.05 ± 0.06	−0.03 ± 0.07	0.18 ± 0.07
Multipara	58	0.04 ± 0.10	0.22 ± 0.11	0.17 ± 0.12	−0.07 ± 0.13
Gestational diabetes mellitus	0.063		0.559	0.351	0.075
Yes	76	−0.10 ± 0.09	−0.15 ± 0.10	−0.02 ± 0.11	0.21 ± 0.12
No	598	−0.28 ± 0.03	−0.21 ± 0.03	−0.13 ± 0.04	−0.02 ± 0.04
Pregnancy 25(OH)D concentration		**0.010**	0.095	0.398	0.236
Suboptimal (<80 nmol/L)	333	−0.34 ± 0.04	−0.26 ± 0.05	−0.15 ± 0.05	−0.04 ± 0.06
Optimal (≥80 nmol/L)	341	−0.19 ± 0.04	−0.15 ± 0.05	−0.09 ± 0.05	0.05 ± 0.06
UCB 25(OH)D concentration	0.149		0.486	**0.003**	0.234
Suboptimal (<80 nmol/L)	369	−0.22 ± 0.04	−0.18 ± 0.04	−0.02 ± 0.05	0.05 ± 0.05
Optimal (≥80 nmol/L)	305	−0.31 ± 0.04	−0.23 ± 0.05	−0.24 ± 0.05	−0.05 ± 0.06

*25(OH)D* 25-hydroxy vitamin D, *UCB* umbilical cord blood

Values are adjusted mean ± SEM. *p* value is in bold when statistically significant

^a^ Adjusted for maternal height and other listed maternal factors as changing covariates

^b^ Two values are missing from analyses

^c^ Adjusted for gestational age

## Discussion

The primary focus of this work was to determine whether 25(OH)D concentration differs between mothers with and without GDM, and whether vitamin D status affects birth size in normal-birth-weight infants. GDM was diagnosed in 11% of mothers in our cohort. Almost all mother-child pairs (97%) were vitamin D sufficient [25(OH)D ≥ 50 nmol/L], and about half of the mother-child pairs had optimal vitamin D status [25(OH)D ≥ 80 nmol/L]. Maternal 25(OH)D concentrations were similar in GDM and non-GDM mothers. Interestingly, pregnancy vitamin D status associated positively with birth weight, but an inverse association was observed between newborn vitamin D status and head circumference at birth.

Lu et al. (2016) concluded in their meta-analysis that maternal vitamin D insufficiency (< 50 nmol/L or <75 nmol/L) was associated with greater risk of GDM [6]. However, they suggested that this applied only in developed countries and when no adjustments for confounders were made [6]. In our study, maternal vitamin D concentrations were similar in non-GDM and GDM mothers, and there was no linear association between vitamin D concentrations and OGTT results. However, we observed that vitamin D deficiency at delivery was more prevalent in mothers with GDM compared with non-GDM mothers, but this was not marked during pregnancy. This finding was possibly confounded by smoking, which was more prevalent in the deficient GDM mothers compared with deficient non-GDM mothers. Nevertheless due to small number of deficient mothers we could not investigate this reliably. In accordance with other studies, our results imply that in a vitamin D sufficient population, the association between 25(OH)D and GDM may not exist [8, 21, 22]. Similarly, Josefson et al. (2016) stated that maternal fasting glucose or GDM status was not associated with pregnancy 25(OH)D, which in their study was on average 93 nmol/L [23].

GDM is a multifactorial disease involving various risk factors, for example lifestyle factors, obesity, rapid weight gain and predisposing genetic factors. Furthermore, some of these factors are related to or co-exist with poor vitamin D status [24, 25], which further increases the challenge when dissecting independent effect. It is possible that in previous studies where no adjustment for confounding factors was performed, the association between 25(OH)D and GDM reflects shared factors such as an unhealthy lifestyle or adiposity [26]. Yet, contrary to many studies, associations between high 25(OH)D and GDM have been reported [27, 28]. Although a biological mechanism between low vitamin D status and diabetes is plausible [29], only a few interventions have been conducted, and these have not proved an effect of vitamin D supplementation on risk of GDM [30].

Maternal vitamin D status associated with birth size: pregnancy 25(OH)D showed a positive association towards birth weight, but UCB 25(OH)D an inverse association with head circumference, while only the inverse association with head circumference was verified with linear model. Harvey et al. (2014) concluded that modest evidence exist for a positive relation between maternal vitamin D status and birth weight [31]. Some earlier studies have shown that severe maternal vitamin D deficiency associates with smaller head circumference at birth [32, 33], yet some have not [34, 35]. In agreement with our findings, others have discovered that mothers with higher vitamin D concentration have infants with smaller head circumference at birth [36, 37]. However, the clinical relevance, if any, of the inverse relationship between maternal vitamin D status and head circumference at birth remains unexplained. It is unknown whether this reflects differences in brain size or in skull bones’ structure, and needs to be explored in future studies. The mean difference in head circumference was 0.22 SD units between the groups with optimal and suboptimal UCB 25OHD. A possible explanation for this might be a U-shaped association between maternal vitamin D concentration and fetal outcomes in a population with sufficient vitamin D status. We have previously suggested the U-shaped association between 25(OH)D concentration and inflammatory biomarkers in cord blood [5].

We have collected a homogenous cohort of Caucasian mothers from the capital region of Finland representing mothers without regular medication and their newborns who were born at term with normal birth weight. In many previous studies, participants had various ethnic backgrounds which could affect both vitamin D status and their risk of GDM [7, 38]. A strength of the present study lies in the recruitment of subjects, which took place in a single hospital, enabled standardised data collection and covered all seasons. However, a multi-centre study might have resulted in a wider variety of socio-economic backgrounds.

A challenge in previous studies on vitamin D status and GDM has been the lack of relevant adjustments, for example adjustments for BMI and smoking status [6]. In the present study, we have systematically investigated confounders between groups, and adjusted for those when applicable. The threshold for defining vitamin D deficiency and the diagnostic criteria for GDM vary between studies, which might affect the results and complicate the comparison of studies. A further limitation is that the OGTT was not performed on all mothers, and a slight possibility exists that the actual prevalence of GDM might be underestimated. Yet, our main results were repeated in a subgroup analysis of only those women to whom an OGTT was performed. In addition, the prevalence of GDM was in accordance with the national statistics [12]. However, in a cross-sectional setting causal relationships cannot be determined.

## Conclusion

In summary, maternal vitamin D concentration was similar in mothers with and without GDM in a vitamin D sufficient population. Furthermore, we found an inverse association between UCB 25(OH)D and infant head circumference. The clinical relevance of this finding remains unsolved and needs to be considered in future studies. Sufficient maternal vitamin D status, specified as 25(OH)D above 50 nmol/L, seems a threshold value, above which the physiological requirements of pregnancy are achieved. Our findings suggest that an adequate maternal vitamin status have been achieved in Finland. However, randomised controlled trials are required in specific risk groups of vitamin D deficiency to clarify if vitamin D supplementation affects the risk of GDM.

## Additional file

Additional file 1: DEQAS July 2014 25-hydroxyvitamin D positive bias. (PDF 655 kb)

## Abbreviations

25(OH)D: 25-hydroxy vitamin D concentration
GDM: Gestational diabetes mellitus
GWG: Gestational weight gain
UCB: Umbilical cord blood
