# Supplementary material for: Maternal vitamin D status, gestational diabetes and infant birth size
Source: BMC Pregnancy Childbirth. 2017 Dec 15;17:420. doi: 10.1186/s12884-017-1600-5 (PMC5732505; doi:10.1186/s12884-017-1600-5)
Supplement: Additional file 1: — DEQAS July 2014 25-hydroxyvitamin D positive bias. (PDF 655 kb) [file 12884_2017_1600_MOESM1_ESM.pdf]

October 23rd, 2014

RE: DEQAS July 2014 25-hydroxyvitamin D positive bias

Dear Valued Customer,

The 25-Hydroxyvitamin DEQAS - July 2014 distribution report indicates that the results obtained with the IDS-iSYS 25-Hydroxy Vitamin D^S assay were outside the ± 25% criteria.

Immunodiagnostic Systems launched an internal investigation by verifying the alignment between the IDS-iSYS 25-Hydroxy Vitamin D^S and the ID-LC-/MS/MS 25(OH)D Reference Method Procedure (RMP) using the single donor serum samples from the Vitamin D Standardization Program (VDSP). The DEQAS July 2014 distribution and our internal serum samples panels were also measured in multiple reagent lots and systems.

We have confirmed a bias in DEQAS - July 2014 distribution. The regression slope between the IDS-iSYS assay and the RMP is slightly higher than previously communicated in the Product Notification NIS2700S/04 (1.06 vs. 1.04); the mean % bias is 12% versus -2%. The bias occurred due to the implemementing of a new internal serum panel preparation with target value slightly higher than the LC-MS/MS value.

From kit lot 2191 and onward, we have adjusted the internal serum panel target value to correct the bias. The summary of IDS-iSYS 25-Hydroxy Vitamin D^S Traceability is enclosed for your references. The results confirm the alignment against the ID-LC-/MS/MS 25(OH)D Reference Method Procedure (RMP).

Immunodiagnostic Systems strives to provide you with products of the highest quality. We value your business and thank you for your continued support. Please contact your local IDS representative if you have any further questions regarding this information.

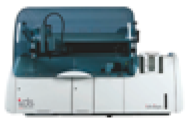

Immunodiagnostic Systems Limited
10 Didcot Way
Boldon Business Park
Boldon - Tyne & Wear
NE35 9PD - UK
Tel: +44 (0) 191 519 6163
Fax: +44 (0) 191 519 0760
Email: techsupport.uk@idsplc.com

Immunodiagnostic Systems France SAS
153 Avenue D'Italie,
75013 Paris – France
Tel: +33 (0) 40 77 04 70
Fax: +33 (0) 1 40 77 04 77
Email: support-technique@idsplc.com

Immunodiagnostic Systems GmbH (IDS GmbH)
Mainzer Landstrasse 49
60329 Frankfurt am Main
Germany
Tel: +49 (0) 69 3085 5025
Fax: +49 (0) 69 3085 5125
Email: techsupport.de@idsplc.com

Immunodiagnostic Systems Nordic a/s (IDS Nordic a/s)
International House,
2300 København S
Center Boulevard 5 - Denmark
Tel: +45 44 84 00 91
Email: techsupport.nordic@idsplc.com

Immunodiagnostic Systems Inc (IDS Inc)
8425 N. 90th Street, Suite #8
Scottsdale, AZ 85258
USA
Tel: 877-852-6190
Fax: 480-836-7437
Email: techsupport.us@idsplc.com

Immunodiagnostic Systems SA
101, rue Ernest Solvay
B 4000 LIEGE, Belgium
Tél (Hotline Francophone): (0)4 229 25 28
Tél (English-Speaking Hotline): (0)4 229 25 27
Fax: +32 (0) 4 252 51 96
Email : support.be@idsplc.com

IDS-iSYS 25-Hydroxy Vitamin D^S (ng/mL) VDSP Traceability

The single donor serum samples (n = 70) with RMP ID-LC-MS/MS 25(OH)D target value ranging from 9.0 – 79.2 ng/mL from the Vitamin D Standardization Program (VDSP) were used to verify the IDS-iSYS alignment in May 2014. The same samples were measured in September 2014 to confirm the assay traceability. From kit lot 2191 onward, we have adjusted the internal serum panel target value to correct the bias.

<table>
<tr><th></th><th>Passing-Bablok regression</th><th>Linear regression</th><th>Statistical summary</th></tr>
<tr><td>Product Notification
NIS2700S/04
May 2014</td>
<td>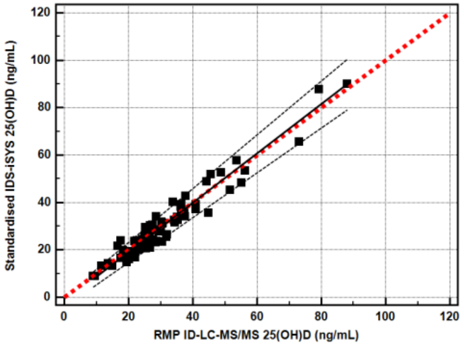
</td>
<td>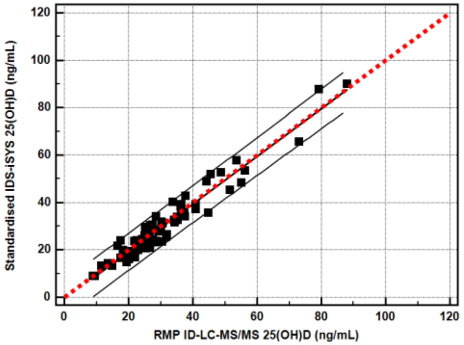
</td>
<td>The Passing-Bablok regression between the IDS-iSYS (y) and the RMP ID-LC-MS/MS (x) is:
IDS-iSYS = 1.04 x (RMP) -1.6 ng/mL
95 % CI. slope: 0.95 to 1.13
95 % CI. intercept: -4.2 to 0.5 ng/mL
Pearson corr. coeff. r: 0.967 (0.948 to 0.980), P<0.0001
Mean %bias: -2.0%
The linear regression equation is:
IDS-iSYS = 1.00 x (RMP) – 0.8 ng/mL
95 % CI. slope: 0.94 to 1.07
95 % CI. intercept: -3.0 to 1.4 ng/mL</td></tr>
<tr><td>Alignment verification
September 2014</td>
<td>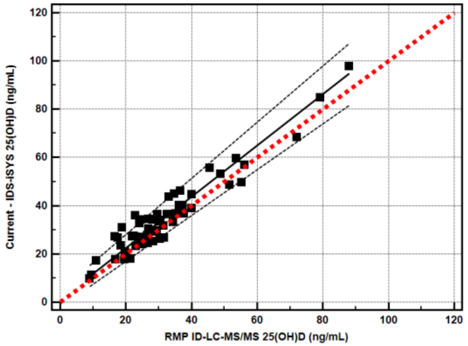
</td>
<td>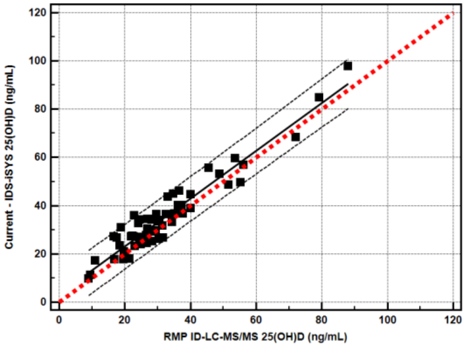
</td>
<td>Although the Passing-Bablok regression yields a slope >1.05, the slope of linear regression is 0.99.
The Passing-Bablok regression is:
IDS-iSYS = 1.06 x (RMP) +1.0 ng/mL
95 % CI. slope: 0.95 to 1.17
95 % CI. intercept: -2.2 to 4.6 ng/mL
Pearson corr. coeff. r: 0.954 (0.932 to 0.970), P<0.0001
Mean %bias: 12.0%
The linear regression equation is:
IDS-iSYS = 0.99 x (RMP) + 3.3 ng/mL
95 % CI. slope: 0.91 to 1.07
95 % CI. intercept: 0.6 to 6.0 ng/mL</td></tr>
<tr><td>Corrected -
October 2014</td>
<td>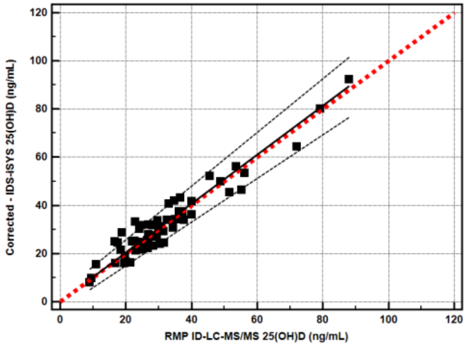
</td>
<td>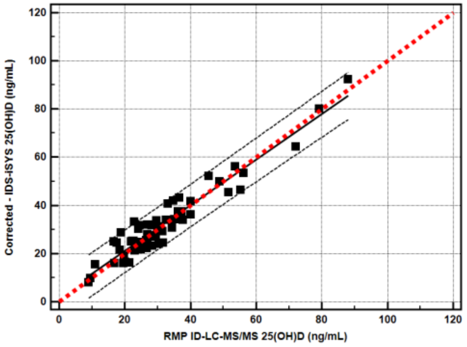
</td>
<td>After the correction, the Passing-Bablok regression yields a slope of 1.02 and the linear regression slope is 0.95. The Passing-Bablok regression is:
IDS-iSYS = 1.02 x (RMP) -0.0 ng/mL
95 % CI. slope: 0.91 to 1.11
95 % CI. intercept: -3.0 to 3.0 ng/mL
Pearson corr. coeff. r: 0.954 (0.932 to 0.970), P<0.0001
Mean %bias: 3.1%
The linear regression equation is:
IDS-iSYS = 0.95 x (RMP) + 2.1 ng/mL
95 % CI. slope: 0.87 to 1.02
95 % CI. intercept: -0.5 to 4.7 ng/mL</td></tr>
</table>

IDS-iSYS 25-Hydroxy Vitamin D^{S} (nmol/L) VDSP Traceability

The single donor serum samples (n = 70) with RMP ID-LC-MS/MS 25(OH)D target value ranging from 23 – 198 nmol/L from the Vitamin D Standardization Program (VDSP) were used to verify the IDS-iSYS alignment in May 2014. The same samples were measured in September 2014 to confirm the assay traceability. From kit lot 2191 onward, we have adjusted the internal serum panel target value to correct the bias.

<table>
<tr><th></th><th>Passing-Bablok regression</th><th>Linear regression</th><th>Statistical summary</th></tr>
<tr><td>Product Notification
NIS2700S/04
May 2014</td>
<td>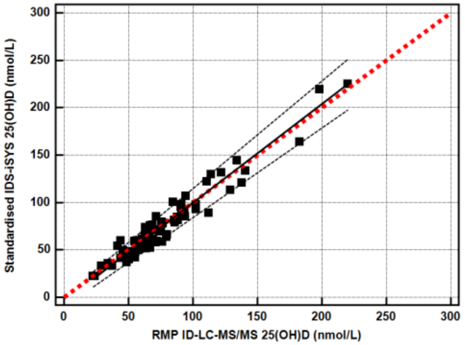
</td>
<td>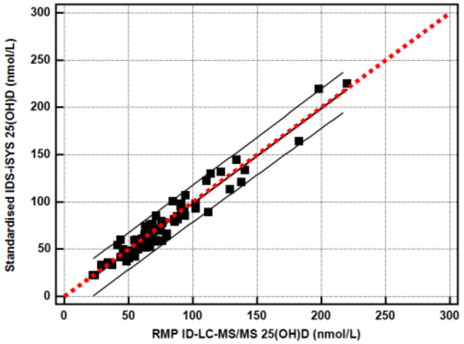
</td>
<td>The Passing-Bablok regression between the IDS-iSYS (y) and the RMP ID-LC-MS/MS (x) is:
IDS-iSYS = 1.04 x (RMP) - 4.0 nmol/L
95 % CI. slope: 0.95 to 1.13
95 % CI. intercept: -10.5 to 1.4 nmol/L
Pearson corr. coeff. r: 0.967 (0.948 to 0.980), P<0.0001
Mean %bias: -2.0%
The linear regression equation is:
IDS-iSYS = 1.00 x (RMP) – 2.0 nmol/L
95 % CI. slope: 0.94 to 1.07
95 % CI. intercept: -7.6 to 3.5 nmol/L</td></tr>
<tr><td>Alignment verification
September 2014</td>
<td>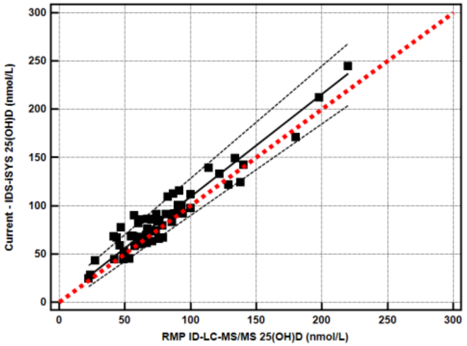
</td>
<td>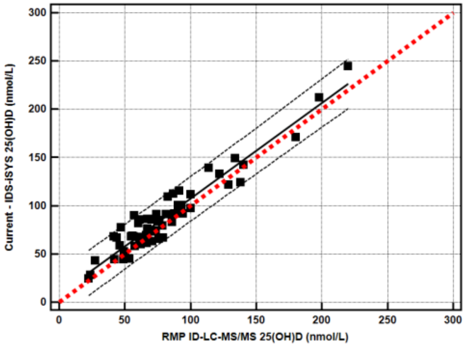
</td>
<td>Although the Passing-Bablok regression yields a slope >1.05, the slope of linear regression is 0.99.
The Passing-Bablok regression is:
IDS-iSYS = 1.06 x (RMP) +2.6 nmol/L
95 % CI. slope: 0.95 to 1.17
95 % CI. intercept: -5.4 to 11.5 nmol/L
Pearson corr. coeff. r: 0.954 (0.932 to 0.970), P<0.0001
Mean %bias: 12.0%
The linear regression equation is:
IDS-iSYS = 0.99 x (RMP) + 8.2 nmol/L
95 % CI. slope: 0.91 to 1.07
95 % CI. intercept: 1.5 to 15.0 nmol/L</td></tr>
<tr><td>Corrected -
October 2014</td>
<td>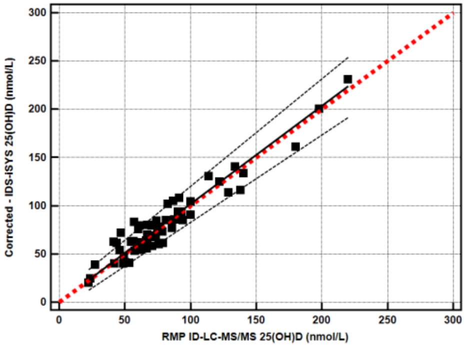
</td>
<td>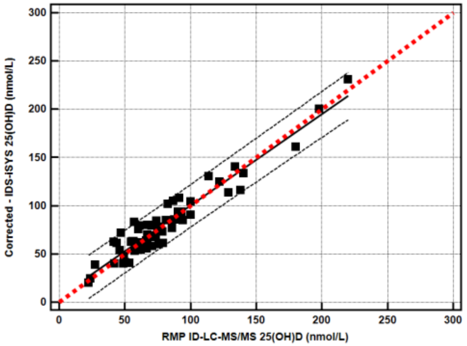
</td>
<td>After the correction, the Passing-Bablok regression yields a slope of 1.02 and the linear regression slope is 0.95. The Passing-Bablok regression is:
IDS-iSYS = 1.02 x (RMP) - 0.0 nmol/L
95 % CI. slope: 0.91 to 1.11
95 % CI. intercept: -7.6 to 8.8 nmol/L
Pearson corr. coeff. r: 0.954 (0.932 to 0.970), P<0.0001
Mean %bias: 3.1%
The linear regression equation is as follow:
IDS-iSYS = 0.95 x (RMP) + 2.1 nmol/L
95 % CI. slope: 0.87 to 1.02
95 % CI. intercept: -0.5 to 4.7 nmol/L</td></tr>
</table>
